# A Mistake in Diagnosis

**Published:** 1899-10

**Authors:** H. C. Gilchrist

**Affiliations:** Nyack, N. Y.


THE
AMERICAN JOURNAL
—OF—
DENTAL SCIENCE.

VOL. XXXIII. BALTIMORE, OCTOBER, 1899. No. 6.

### Selections.

ARTICLE I.

### A MISTAKE IN DIAGNOSIS.

H. C. GILCHRIST, D. D. S., NYACK, N. Y.

As we all are liable to make mistakes at times in our diagnosis of different cases, I report this incident of office practice as it may benefit some one in the future who might have a similar case.

Miss T——, a young lady of about twenty years of age, came to me for consultation regarding a fistulous opening in the right superior maxilla just back of the of the second molar. She stated that there had been a slight flow of pus for about three months previously accompanied with neuralgia pains which had been a great annoyance to her. Upon examination I found I could pass a probe up through the fistulous tract for about one inch before I came in contract with any obstruction.

I diagnosed the case as encysted wisdom tooth due to lack of room for eruption and stated that it would need to be removed before the jaw would get well, but she was a little in doubt as to my opinion and decided to wait awhile before submitting to an operation.

Her home being in Florida, I did not see her again for one year, after which she came to my office accompanied by a physician, who stated that something must be done for her, as she was very much reduced in health and quite worried in mind.

Upon questioning her I found that she had been through the hands of several physicians and dentists at her home, all of whom had diagnosed the case as one of necrosis of the jaw bone, except one, and he said that it was a cancerous formation and that the only remedy was an external operation.

But inasmuch as she was a pretty and prepossessing young woman she had hesitated to have the operation performed until she had seen me once more, whereupon I made another examination with her physician, and again I diagnosed it as an unerupted wisdom tooth, but he seemed to think that it was necrosed bone.

I gave her gas and extracted her second molar tooth, then with my lancet I opened the gum from the socket of the extracted tooth to the fistulous opening, cutting down to the bone of the alveolar process. I then ordered the wound to be kept packed with antiseptic gauze for one week, changing the gauze each day. At the end of that time I removed the gauze and upon an examination with the mouth mirror I discovered the glistening surface of a tooth. The attending physician then admitted that I was correct in my diagnosis after all.

I administered gas again and undertook to extract the tooth, but much to my amazement just as I attempted to grasp the tooth it disappeared from my view; in feeling for it with a probe I found a pus cavity about half an inch in diameter and the tooth at the bottom of it.

The patient being somewhat exhausted I deferred the removal of the tooth until another time. The next day her physician in probing in the cavity dislodged the tooth and it fell down in her mouth. She came to me with it in her hand and with a smile upon her face she expressed her gratitude for what had been done for her.

I kept the cavity open with a strip of antiseptic gauze so as to allow the healthy granulations to deposit at the bottom of the cavity and directed her to use Listerine as a mouth wash. This was kept up for about three weeks, when the wound healed nicely, leaving the jaw and the surrounding parts in a perfectly healthy condition.—*Items of Interest.*

